# The Screening of Aptamers and the Development of a Colorimetric Detection Method for the Pesticide Deltamethrin

**DOI:** 10.3390/s25072060

**Published:** 2025-03-26

**Authors:** Caixia Wu, Wenwei Li, Jiafu Wang, Sheng Li

**Affiliations:** 1 Key Laboratory of Plant Resource Conservation and Germplasm Innovation in Mountainous Region (Minister of Education), Department of Life Science/Institute of Agro-Bioengineering, Guizhou University, Guiyang 550025, China; wucaixia553898@163.com (C.W.); liwenwei516@163.com (W.L.); jfwang@gzu.edu.cn (J.W.); 2 Department of Medical Technology, Chongqing Medical and Pharmaceutical College, Chongqing 400000, China

**Keywords:** deltamethrin, aptamer, Capture-SELEX, colorimetric sensor, AuNPs

## Abstract

Deltamethrin (Del), a widely utilized pyrethroid pesticide, exhibits significant risks to human health due to its persistent environmental residues. This study aims to develop an efficient sensing detector for rapid Del detection through aptamer-based recognition. A modified Capture-SELEX strategy successfully identified Del-1, a high-affinity DNA aptamer demonstrating specific binding to Del with a dissociation constant (Kd) of 82.90 ± 6.272 nM. Molecular docking analysis revealed strong intermolecular interactions between Del-1 and Del, exhibiting a favorable binding energy of −7.35 kcal·mol^−1^. Leveraging these findings, we constructed a colorimetric detector using gold nanoparticles (AuNPs) and poly dimethyl diallyl ammonium chloride (PDDA)-mediated aggregation modulation. The sensing detector employed dual detection parameters: (1) a characteristic color transition from red to blue and (2) a quantitative ∆A650/A520 ratio measurement. This optimized system achieved a detection limit of 54.57 ng·mL^−1^ with exceptional specificity against other competitive pesticides. Practical validation using spiked fruit samples (apples and pears) yielded satisfactory recoveries of 74–118%, demonstrating the sensor’s reliability in real-sample analysis. The developed methodology presents a promising approach for the on-site monitoring of pyrethroid contaminants in agricultural products.

## 1. Introduction

Deltamethrin (Del) is the primary representative of pyrethroid insecticides, and is widely used in the control of coleoptera, lepidoptera, and dipteral pests in agriculture and acts by causing both gastric toxicity and poisoning by touch [1,2]. As traditional highly toxic pesticides, such as organophosphorus pesticides, are gradually being banned or restricted, the production and use of Del are growing rapidly, and its environmental residue levels are increasing year by year. Its potential ecological risks and hazards to human health have attracted considerable attention on a global scale [3,4]. Current studies have shown that Del is a widely present residue in food, the atmosphere, and water in indoor and outdoor environments, and human health may be harmed through long-term exposure to the skin, mouth, or respiratory tract [5,6]. The World Health Organization’s International Agency for Research on Cancer (IARC) added Del to its list of carcinogens in 2017 [7]. Many countries have stipulated the maximum residue limit (MRL) of Del in food, and China has specified that its MRL is 0.01–10 mg·kg^−1^ [8]. Given Del’s residual toxicity, there is an urgent need to develop a rapid, selective, and sensitive method for the detection of Del residues in food and the environment.

Currently, the main detection methods for deltamethrin in food and the environment are chromatographic techniques and immunoassays based on enzyme-linked immunosorbent assay (ELISA). Chromatography is not suitable for the on-site detection of many samples due to the complexity of the pre-treatment process, expensive detection equipment, and the need for specialized instrumental analysts [9,10]. On the other hand, antibody-based detection has many limitations. Since deltamethrin is a non-immunogenic small-molecular compound, antibodies prepared from semi-antigens formed by structural modification tend to have high cross-reactivity (36.93%) [11]. At the same time, antibody preparation requires complex animal immunization procedures lasting 6–12 weeks, and monoclonal antibodies can cost up to USD 10 000 [12]. Antibody storage conditions are also environmentally demanding, with approximately 83% of field-tested antibodies reported to deteriorate at >40 °C and pH < 5 [13]. Therefore, screening and discovering new Del-recognition molecules and establishing more efficient and economical real-time detection methods are of great significance in pesticide residue detection in food and the environment.

Aptamers are a class of single-stranded oligonucleotides (DNA or RNA) consisting of 25–80 bases [14]. The aptamer can self-fold through mutual bonding, hydrogen bonding, electrostatic interaction, etc., and then form stable three-dimensional spatial structures, such as stem rings, hairpins, G-quadruplexes, etc., which can bind to target molecules with high specificity and high affinity [15,16]. When compared with antibodies, aptamers have the advantages of high stability, easy modification, good repeatability, simple synthesis, and high cost-effectiveness [17]. Therefore, aptamers are often used as recognition probes to detect target molecules. Aptamers that bind to various substances, such as proteins, toxins, cells, and antibiotics, have been widely screened and used [18]. The Capture-SELEX strategy is widely used to screen aptamers of small molecules that are difficult to attach to vectors [19]. Based on the Capture-SELEX strategy, an auxiliary sequence is introduced by improving its design. The 3′ end of the auxiliary sequence can be paired with random ssDNA library bases, and the 5′ end is labeled with biotin, so that the random ssDNA library can be attached to streptavidin beads, overcoming the problem of excessively long adaptor length and improving the screening efficiency. This improved Capture-SELEX strategy has been reported to successfully select aptamers that bind to lambda-cypermethrin [20], clothianidin [21], dimethyl cadmium ion [22], paraquat [23], and ethyl carbamate [24]. Del, as a small-molecular insecticide, also does not have active molecules that can be attached to the vector, and it is speculated that this improved Capture-SELEX screening strategy can also be used to obtain specific nucleic acid aptamers of Del; however, no relevant studies on this topic have been reported so far.

In addition to using aptamers as probe identification targets, related biosensors must also be used to amplify the recognition signal for easy detection. Among the aptamer biosensors, AuNP-based colorimetric sensors have the advantages of low cost, easy operation, and speed [25,26]. AuNPs are usually evenly dispersed in the solution and appear as a red transparent colloid [27]. When the salt concentration is high, the AuNPs aggregate to form larger particles or aggregates, and the color of the colloidal solution gradually turns blue [28]. Therefore, in this study, we propose developing a rapid and straightforward colorimetric assay using the Del aptamer as the recognition element and the degree of aggregation of AuNPs induced by cationic compounds as the detection signal.

In this study, we used the improved Capture-SELEX strategy to enrich single-stranded ssDNA that specifically binds Del from random DNA libraries. Then, we found the aptamer with the strongest binding to Del through high-throughput sequencing, sequence characterization, and Del detachment constant (Kd) analysis. Finally, a new colorimetric detection method for Del was developed using the aptamer as the recognition molecule and the cationic polymer-mediated gold aggregation as the detection signal. The method has the advantages of fast sensitivity, identification with the naked eye, and low cost.

## 2. Materials and Methods

### 2.1. Instrumentation and Reagents

The following instruments and reagents were utilized in this study: RTE7 Thermostatic Water Bath (Thermo Fisher Technology, Waltham, MA, USA); PTC-PCR Instrument (MJ RESEARCH, Santa Rosa, CA, USA); PowerPC Miniature Electrophoresis Apparatus (BIO-RAD Corporation, Hercules, CA, USA); Complete Wavelength Enzyme Label Analyzer (Beijing Putian Xinqiao Technology Co., Ltd., Beijing, China); Ultra-microspectrophotometer K5600 (Beijing Kaiao Technology Development Co., Ltd., Beijing, China); ssDNA (5′-ACCGACCGTGCTGGACTCT-N30-AGTATGAGCGAGCGTTGCG-3′), Primer F (5′-ACCGACCGTGCTGGGACTCT-3′), Primer R (5′-Biotin-CGCAACGCTCGCTCATACT-3′); biotin-labeled sequence P (5′-Biotin-CGCAACGCTCGC-3′); non-denaturing gel kit, PCR amplification kit, and DNA Marker (Shanghai Bioengineering Co., Ltd., Shanghai, China); Deltamethrin (Del) and its competitive pesticides (Cypermethrin, Fenpropathrin, Carbendazim, Profenofos, Glyphosate, Paraquat), AuNPs, poly (poly dimethyl diallyl ammonium chloride) (PDDA), PBS Screening buffer, and MOPS Buffer (Hubei Sawai Biotechnology Co., Ltd., Ezhou, Hubei, China); drugs for electrophoresis (5 × TBE, bisacrylamide, acrylamide, ammonium persulfate, AgNO_3_); and streptavidin magnetic beads (1 μm particle size, 10 mg·mL^−1^) (Anhui Angptomai Biotechnology Co., Ltd., Hefei, Anhui, China). All the chemical reagents were commercially available and analytically pure, and the experimental water was sterile double-steamed water.

### 2.2. Screening of Del Aptamer

#### 2.2.1. The Strategy for Screening Del Aptamers

**Figure 1:**
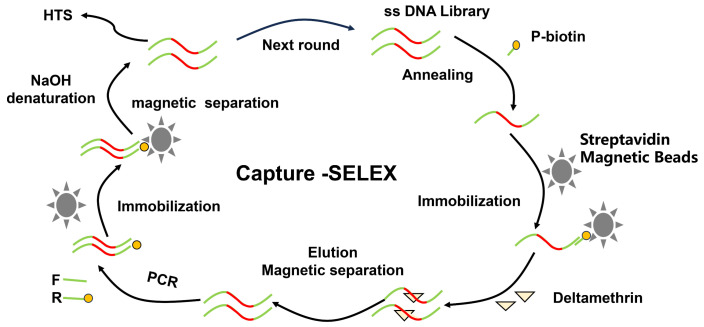
The Capture-SELEX strategy roadmap for screening aptamers against Del.

Figure 1 shows the optimized Capture-SELEX strategy used in this study. The general steps were to immobilize a random ssDNA library on streptavidin magnetic beads with auxiliary sequences, add a certain concentration of the target molecule Del for co-incubation, and then collect the nucleic acid sequences bound to Del for PCR amplification. The PCR amplification products were denatured and immobilized again as ssDNA libraries on streptavidin magnetic beads for the next round of co-incubation with Del. After such rounds of cycling, nucleic acid sequences that bind specifically to Del were enriched. We utilized this enrichment system to screen for aptamers with a greater affinity for Del by gradually decreasing the concentration of Del during each round of incubation.

#### 2.2.2. Fixing of Random ssDNA

A 1 nmol random ssDNA library was mixed with 2 nmol auxiliary sequence P, denatured at 95 °C for 5 min, cooled at 4 °C, and incubated at 30 °C for 60 min. P was paired with 12 base sequences during incubation at the 3′ end of the random ssDNA library. Subsequently, streptavidin magnetic beads were added to the mixture and incubated at 30 °C for 120 min to immobilize the ssDNA library on the magnetic beads with biotin-affinity interaction. Next, the beads were washed three times with PBS buffer to remove unbound random libraries and P sequences. Finally, the washed magnetic beads were re-suspended in the PBS buffer.

#### 2.2.3. Elution of Affinity ssDNA Sequences

A certain amount of Del was added to a 1.5 mL centrifuge tube containing a magnetic bead–ssDNA library complex, and the mixture was shaken for 5 min to ensure adequate mixing. Next, incubation at 30 °C for 2 h was performed to facilitate the interaction of Del with random ssDNA on the surface of the magnetic beads, resulting in a conformational change in the DNA sequence, which was dislodged from the magnetic beads. After incubation, the centrifuge tube was placed on the magnetic rack for 3 to 5 min, the supernatant (containing nucleic acid sequences bound to Del) was collected, and the ssDNA concentration was determined using an ultra-micro spectrophotometer (Beijing Kaiao Technology Development Co., Ltd., Beijing, China). To increase the screening pressure, the concentration of Del was gradually reduced from 250 μg·mL^−1^ to 1 μg·mL^−1^ in subsequent Capture-SELEX screening.

#### 2.2.4. PCR Amplification and Electrophoretic Characterization

The supernatant collected in each round served as the amplification template. The 50 μL amplification system consisted of a 25 μL template, 1 μL 5 μmol·L^−1^ forward primer F, 1 μL 5 μmol·L^−1^ reverse primer R, 20 μL 2X PCR Master, and 3 μL ddH_2_O mixed and shaken. The amplification conditions were as follows: pre-denaturation at 94 °C for 3 min, 22 rapid cycles (denaturation at 95 °C for 30 s, annealing at 60 °C for 30 s, extension at 72 °C for 30 s), and extension at 72 °C for 5 min. Each round of PCR amplification products was identified using electrophoresis in a 12% non-denaturing PAGE gel.

#### 2.2.5. Preparation of the Next ssDNA Library

The amplification product was mixed with 100 μL of streptavidin magnetic beads, and then 350 μL of 4 M NaCl was added, and this mixture was incubated at 30 °C for 45 min. At the end of the incubation, the supernatant was discarded, 100 μL of 40 mM NaOH was added, and the supernatant was extracted by mixing with a pipette for 60 s and letting it stand for 5 min on a magnetic rack. The affinity ssDNA was obtained by neutralizing NaOH in the supernatant with 1.9 μL 1 M HCI.

### 2.3. High-Throughput Sequencing and Secondary Structure Prediction of Del Aptamers

The final round of PCR amplification products was subjected to high-throughput sequencing (HTS) on an Illumina MiSeq machine. According to the aptamer frequency, the top 15% of affinity ssDNA sequences were selected for homology analysis and structural study. The phylogenetic tree of 15 affinity sequences was constructed with homology analysis using the MEGA 11 software, and the secondary structure was analyzed and predicted using the Mfold 3.6 software (http://www.mfold.org/), 24 November 2024.

### 2.4. Affinity and Specificity Analysis of Del Aptamers

Candidate aptamers with different concentrations (50–500 nmol·L^−1^) were fixed on streptavidin magnetic beads, and Del (50 μg·mL^−1^) was added. After incubation at room temperature for 2 h, the supernatant was extracted, and the ssDNA concentration of the supernatant was measured using an ultra-micro spectrophotometer. Origin was used for nonlinear fitting, and the Kd values of the candidate aptamer pairs of Del were derived using the formula Y=BmaxX/(Kd+X); where X is the concentration of the incorporated candidate aptamer and Y is the concentration of the candidate aptamer bound to Del. The aptamer was fixed on streptavidin magnetic beads, and the same concentration of Del and other competing pesticides (Cypermethrin, Fenpropathrin, Carbendazim, Profenofos, Glyphosate, Paraquat) was added. After complete incubation for 2 h, the concentration of ssDNA in the eluent was determined to assess the binding specificity of the aptamer. The tertiary structure of the Del molecule was obtained using the Chem3D15.1 software package.

### 2.5. Molecular Docking

We used a molecular docking approach to elucidate the binding mechanism of aptamers to Del pesticide molecules. The output format file was obtained using the secondary structure of Vienna, the online tertiary structure of the adapter body. The pdbqt files of aptamers and Del were generated and docked using the AutoDock Tools 1.5.7 software to analyze the interaction and potential binding sites between aptamers and Del. Finally, the docking results were visualized using the PyMOL 2.6.0 software.

### 2.6. Construction of AuNPs for Colorimetric Detection of Del

A 200 μL volume of the AuNP solution and 8 μL of the Del-1 aptamer were added to a 1.5 mL centrifuge tube, mixed, and incubated for 10 min at room temperature. Then, 2 μL Del solution was added and incubated for 2 h at room temperature. At the end of the incubation, 220 μL of PDDA solution was added to bring the total system to 500 μL, and the deficient system was supplemented with MOPS buffer. After the samples were incubated at room temperature for 60 min, the absorbances of A520 and A650 were measured using an enzyme marker, where the size of A650/A520 indicates the degree of AuNPs. The blank control group was used with double-distilled water instead of Del, and ∆A650/A520 = A650/A520 – A′650/A′520 was used as the sensing colorimetric signal. The specificity of the prepared colorimetric method was verified with Del analogs (Cypermethrin, Fenpropathrin, Carbendazim, Profenofos, Glyphosate, Paraquat).

### 2.7. Testing of Actual Samples

Fresh apples and pears purchased from a fruit store were used as the test samples for this experiment. The apples and pears were washed four times in distilled water and placed in air vents to dry. Then, 20 g of apples and pears were taken and ground into a homogenate. The homogenate was transferred to a conical bottle and set aside. Then, 50 mL methanol solution and Del standard solution with a final concentration of 500 ng·mL^−1^ and 1000 ng·mL^−1^, respectively, were added, and the mixture was ultrasonically treated for 2 h. At the end of the sonication process, the mixture was centrifuged at 8000 rpm for 15 min. The obtained supernatant was filtered through a 0.22 μm ultrafiltration membrane, and 100 μL of the filtrate was taken, blow dried with nitrogen, and dissolved with 100 μL methanol for colorimetric detection.

## 3. Results

### 3.1. Screening of Del Aptamers

**Figure 2:**
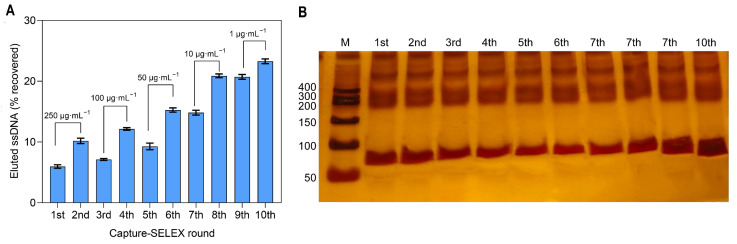
(**A**) The elution rate of the obtained affinity sequences for Del in each round. The concentration of Del in each round of the screening system: 250 μg·mL^−1^ for 1st–2nd rounds, 150 μg·mL^−1^ for 3rd–4th rounds, 50 μg·mL^−1^ for 5th–6th rounds, 10 μg·mL^−1^ for 7th–8th rounds, and 1 μg·mL^−1^ for 9th–10th rounds. (**B**) The characterization of polymerase chain reaction (PCR) products using polyacrylamide gel electrophoresis (PAGE) after eleven rounds of systematic evolution of ligands with exponential enrichment (SELEX) selection.

In this study, the aptamer with a high affinity for Del was obtained by gradually reducing the incubation concentration of the target. The number of rounds of continued screening was assessed based on the elution rate (amount of ssDNA eluted/amount of ssDNA immobilized on magnetic beads) of each round of screening of libraries. We conducted a total of 10 rounds of screening (Figure 2A). The first two rounds were incubated with a high concentration of Del (250 μg·mL^−1^) and a random library to increase the number of sequences with affinity for Del. During subsequent rounds of screening, we gradually reduced the incubated Del concentration from 150 μg·mL^−1^ to 1 μg·mL^−1^. Although the concentration of Del decreased with incubation, the elution rate of Del binding affinity sequences gradually increased, which indicated that Del binding affinity sequences were continuously enriched. During the first 10 rounds of screening, the elution rate of affinity sequences reached the maximum value of 23.28 ± 1.56% in the 10th round. It is generally believed that the elution rate of affinity sequences is above 20%, the enriched sequences have high affinity and binding specificity, and too many enrichment rounds will lead to the “submergence” or loss of high affinity sequences due to non-specific amplification in the PCR process [19,29]. Therefore, we terminated the screening process after the 10th enrichment round.

To verify the affinity sequence size of the affinity enrichment, non-denatured PAGE gel electrophoresis was performed for each of the 10 rounds of PCR amplification and purification of the products (Figure 2B). The products after 10 rounds of PCR may be impure due to primer dimerization, non-specific amplification, contamination, etc., and all of them showed non-specific bands. The target bands of each round were between 50 bp and 100 bp, which was in line with the library design range.

### 3.2. Sequence Analysis of Del Aptamers

The ssDNA obtained using PCR was high-throughput-sequenced. According to the sequencing frequency, the top 15 ssDNA sequences with high affinity were selected as candidate aptamers for Del, and named Del-1–Del-15. Base composition analysis showed that more than 90% of the sequences were “G” at the third base, and about 60% of the sequences were “C” at the fifth base, which may be the binding site of Del. In addition, almost all sequences contained continuous “GGG” bases in the random region, and most of the sequences had a high GC content (Table 1), suggesting that these candidate sequences have a relatively stable secondary structure. GC bases in these sequences may be the key to promoting Del binding to affinity sequences. Gibbs free energy (ΔG) is an essential factor in evaluating the stability of aptamers, and the lower the ΔG, the higher the stability of the secondary structure [30]. Table 1 summarizes the ΔG values of the 15 affinity sequences. The ΔG of Del-1, Del-3, Del-7, Del-12, and Del-19 sequences was lower than −10.0 kJ·mol^−1^, indicating that the secondary structure formed by these sequences is relatively stable, and it is speculated that the combination of these sequences with Del is relatively strong.

**Figure 3:**
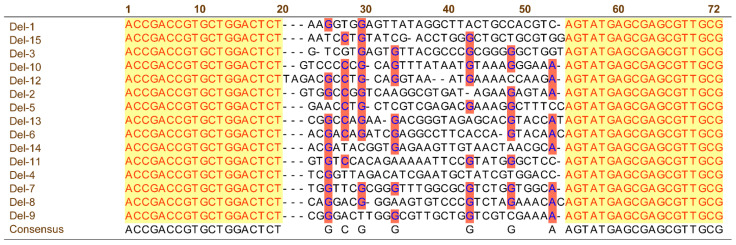
The sequence comparison plot of the affinity sequences of Del (yellow color represents 19 fixed bases; red color represents the same bases appearing at the same position in the affinity sequence).

Based on the number of stem loops and structural similarity, we categorized the above 15 candidate aptamers into four groups (Figures S1–S4). The secondary structures of two aptamers in the first group all contain three rings of similar sizes, and the stem ring of the secondary structure contains a conserved sequence, “AGC”. The secondary structure of nine aptamers in the second group contains four rings, and the composition of the Del-1 and Del-10 stem rings contains the conserved sequence “AUG”. In the other two groups, the secondary structure of the sequence is also dominated by the stem ring structure and the hairpin structure. These structural features suggest that the stem ring and hairpin structures in the affinity sequence may be the recognition sites of Del. The homology of the 15 affinity sequences was analyzed using the MEGA software, and the 15 affinity sequences were classified into four families (Figure 3 and Figure S5). According to the frequency, homology, ΔG, and secondary structure characteristics of 15 affinity sequences, Del-1 was selected as the candidate aptamer to verify affinity and specificity.

### 3.3. Kd Value Determination and Specificity Analysis of the Aptamer

**Figure 4:**
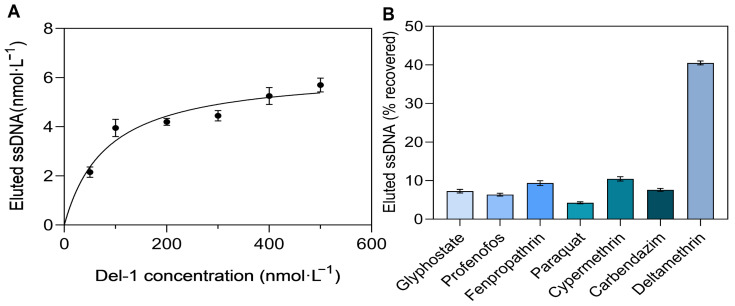
(**A**) The identification of the binding affinity (Kd value) of Del-1 candidate aptamers to Del; (**B**) selectivity of Del-1 to Del. The quantity of each target was considered as 50 μg·mL^−1^.

The Del-1 aptamer was fixed on streptavidin magnetic beads, and the affinity of the Del-1 aptamer was detected. Elution was performed with 50 μg·mL^−1^ Del according to the screening procedure, and the concentration of aptamer in the eluate was determined. Finally, Origin performed a nonlinear fit to calculate its Kd value. As the concentration of Del-1 increases, the elution concentration of Del-1 increases and eventually reaches saturation (Figure 4A). The Kd value of Del-1 was 82.90 ± 6.272 nM, indicating that it has a high affinity for Del. This may be related to covalent bonding interactions such as hydrogen bonding, electrostatic interactions, hydrophobic interactions, base stacking, and van der Waals forces [31]. The specificity of Del-1 was further studied. The elution rate of Del-1 for Del was more than 40% (Figure 4B), which was much higher than that of the other six pesticides (Cypermethrin, Fenpropathrin, Carbendazim, Profenofos, Glyphosate, Paraquat), suggesting that Del-1 has a strong binding specificity for Del.

### 3.4. Del- and Aptamer-Binding Mechanism

**Figure 5:**
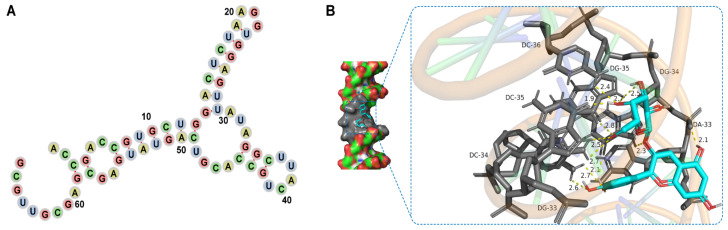
(**A**) Secondary structure of Del-1. (**B**) Ten molecular docking results of Del-1 and Del.

The secondary structure of Del-1 was simulated using the Mfold software (Figure 5A), and the structure consists of two rings of similar sizes. In the macrocycle, the “G” base accounts for 32% of the content, which is presumed to be involved in the binding of Del. The binding sites of Del-1 and Del in space were further simulated using the molecular docking software. Molecular docking showed that the small molecules of Del were bound by hydrophobic action in the hydrophobic cavity of the three-dimensional structure of Del-1, and they were close to the side chain bases of A-33, G-34, G-35, and C-36 (Figure 5B). In addition, Del co-forms hydrogen bonds with Del-1 and further consolidates the binding by crossing these hydrogen bonds. Molecular docking results showed that the binding free energy of Del and Del-1 was as low as −7.35 kcal·mol^−1^, and studies showed that the binding energy was less than −7.0 kcal·mol^−1^, indicating a high binding affinity [32].

### 3.5. The Principle and Feasibility Analysis of the Colorimetric Method

**Figure 6:**
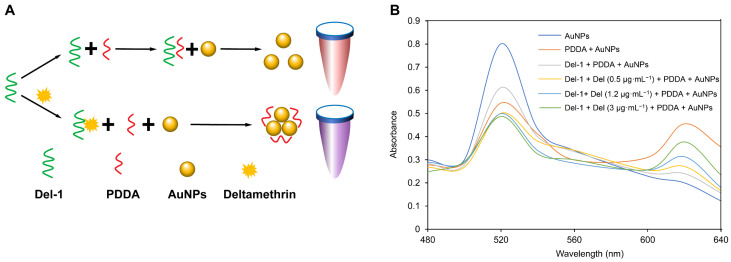
(**A**) Schematic diagram of Del colorimetric sensor; (**B**) absorption spectra of different samples. Sample 1: AuNPs; sample 2: PDDA + AuNPs; sample 3: Del-1 + PDDA + AuNPs; sample 4: Del-1 + Del (0.5 μg·mL^−1^) + PDDA + AuNPs; sample 5: Del-1 + Del (1.2 μg·mL^−1^) + PDDA + AuNPs; sample 6: Del-1 + Del (3 μg·mL^−1^) + PDDA + AuNPs.

In this study, the Del-1 aptamer was used as the recognition molecule, the aggregation of AuNPs regulated by PDDA was selected as the sensing signal, and Del was detected using the colorimetric method. The complete detection principle is shown in Figure 6A. In the absence of Del, PDDA can form a “duplex” structure with Del-1 through electrostatic adsorption, and when AuNPs in the system are in the dispersed state, the solution will be red in color and exhibit its maximum absorption peak at 520 nm (Figure 6B and Figure S6). When Del was present, it preferentially interacted with the Del-1 aptamer with high affinity to form a complex, and PDDA induced the aggregation of AuNPs, gradually causing the whole sensing system to turn blue–purple. The absorption peak appeared at 650 nm, and tended to increase with the Del concentration (Figure 6B and Figure S6). To visually explore the feasibility of the colorimetric strategy, the UV absorption spectrograms were measured under different experimental conditions, and the color of the solutions was recorded. When 0.5 μg·mL^−1^, 1.2 μg·mL^−1^, and 3 μg·mL^−1^ of Del were present in the colorimetric system, the solution system gradually changed from red to blue at the beginning. The degree of change in color and absorption spectra was positively correlated with the concentration of Del (Figure 6B and Figure S6). The results indicated that the colorimetric strategy was feasible in Del detection.

### 3.6. The Optimization of the Conditions of the Colorimetric Method

**Figure 7:**
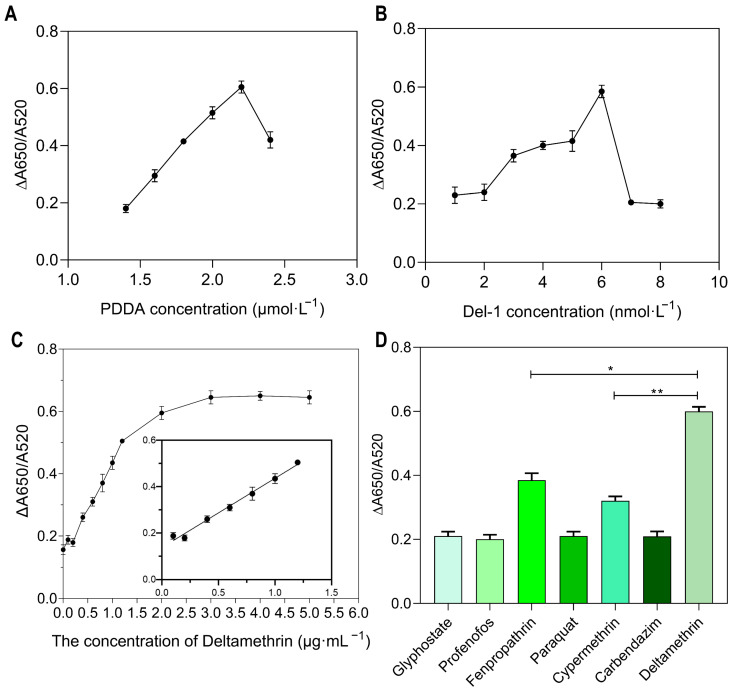
(**A**) The effect of PDDA concentration on the colorimetric system; (**B**) the effect of Del-1 aptamer concentration on the colorimetric systems; (**C**) fitting curves of the detection signal of the Del-1 aptamer detection system at Del concentrations of 0.1 μg·mL^−1^, 0.2 ug·mL^−1^, 0.4 ug·mL^−1^, 0.5 ug·mL^−1^, 0.7 ug·mL^−1^, 0.8 ug·mL^−1^, 1.0 ug·mL^−1^, 1.2 ug·mL^−1^, 2.0 ug·mL^−1^, 3.0 ug·mL^−1^, 4.0 ug·mL^−1^, and 5.0 ug·mL^−1^. (**D**) Signal changes in the whole system after adding 2 μg·mL^−1^ of different pesticides (* *p* < 0.05; ** *p* < 0.001).

Since the overall colorimetric detection signal was determined by the aggregation of AuNPs and correlated with the concentrations of PDDA and Del-1, the concentrations of PDDA and Del-1 were further optimized. We investigated the effect of PDDA concentration on this colorimetric system. The ΔA650/A520 value increased with the increasing PDDA concentration (Figure 7A). When the PDDA concentration was increased to 2.2 μmol·L^−1^, the colorimetric detection signal reached a maximum value of ∆A650/A520 = 0.61. Then, the concentration of Del-1 was optimized at a fixed concentration of 2.2 μmol·L^−1^ PDDA, and the effect of the aptamer concentration on the value of ∆A650/A520 was studied (Figure 7B). When the aptamer concentration was 6 nmol·L^−1^, ∆A650/A520 = 0.58 was the maximum value, and it was either too high or too low; aptamer usage affected the aggregation of AuNPs by PDDA. Therefore, the optimal concentrations of PDDA and Del-1 in this study were 2.2 μmol·L^−1^ and 6 nmol·L^−1^, respectively.

### 3.7. Performance Analysis of the Colorimetric Method

The effects of different concentrations of Del on the ΔA650/A520 values of this colorimetric system and the color of the system were investigated at the optimal concentrations of PDDA and Del-1. As the concentration of Del increased, the system’s color gradually turned red. When the Del concentration was 1.2 μg·mL^−1^, the color of the system changed from dark red to blue (Figure 7C and Figure S7). The ΔA650/A520 values had a good linear relationship with the Del concentration, in the range of 0.1–1.2 μg·mL^−1^, and the linear regression equation was y = 0.2913x + 0.1438 (R^2^ = 0.9800). The Limit of Detection (LOD) of the method was calculated to be 54.57 ng·mL^−1^ based on the 3σ/slope, which is lower than the MRL (200 ng·mL^−1^) of Del defined by China [8]. Therefore, the colorimetric sensor proposed in this study can also be used for the quantitative detection of Del in the micro range.

To assess the specificity of the colorimetric method for the detection of Del, six other common pesticides (Cypermethrin, Fenpropathrin, Carbendazim, Profenofos, Glyphosate, and Paraquat) were tested for their effect on this colorimetric system. The ΔA650/A520 value (0.61) of Del was higher than that of the other six pesticides, indicating that the assay had a reasonable specificity (Figure 7D). We also observed that the ΔA650/A520 values produced by Cypermethrin and Fenpropathrin were about 0.4, which is higher than those of other pesticides except Del, which may be because they belong to the pyrethroid pesticide group. The structural difference between these pesticides and Del is smaller than that of the other four pesticides, suggesting that the aptamer is specific to pyrethroids, with Del as its strongest target.

### 3.8. Detection of Actual Samples

We chose pears and apples as the test samples to verify the detection performance of Del-1 aptamer in actual samples. After the samples were ground for homogenization, the Del standard solution was added to adjust the final concentration of Del in the sample to 500 ng·mL^−1^ and 1000 ng·mL^−1^. The difference in absorbance ΔA650/A520 between the blank and experimental groups was determined under optimal experimental conditions, and the results are shown in Table 2. The recovery of Del was 74–118%, and the relative standard deviation (RSD) was 0.13–6.12%. It is shown that this method can be used to detect Del in real samples. In addition, the performance of the proposed colorimetric sensor for Del was compared with previous analytical methods (Table 3). Compared with GC-FID, HPLC, SERS, ELISA, and electrochemical methods, this method does not require special instrumentation, and the results can be judged by the naked eye, which is convenient, cost-effective, and takes relatively less time for the detection of a single sample. Meanwhile, the detection limit of this method is lower than that of the colorimetric method, which uses 2-mercapto-6-nitrobenzothiazole as the substrate for the chemical reaction. Therefore, in this study, the colorimetric sensor constructed with the nucleic acid aptamer is simple, rapid, economical, and has a promising application in the detection of Del.

## 4. Conclusions

We obtained a Del-1 aptamer with high affinity and specificity for Del using the Capture-SELEX technique, and the dissociation constant (Kd) of Del-1 was 82.90 ± 6.272 nM. Molecular docking simulations showed that Del is close to Del-1 side chain bases, i.e., A-33, G-34, G-35, and C-36, with a minimum free binding energy of −7.35 kcal·mol^−1^. Next, a visual colorimetric detector for the detection of Del was successfully developed based on the color change in the PDDA-aggregated nanogold solution using Del-1 as the recognition element and the ΔA650/A520 values as the sensing signals. Under the best-optimized conditions, ΔA650/A520 showed a good linear relationship with Del concentration in the range of 0.1–1.2 μg·mL^−1^, with an LOD of 54.57 ng·mL^−1^ and recoveries of 74–118% in the reagent samples. The colorimetric sensor in this study saves a lot of time, reduces cost, and has high sensitivity and selectivity compared with other sensors. This study demonstrates the feasibility of utilizing Del aptamers for food safety control and opens up new avenues for the future development of Del molecular probes and detection technologies.

## Figures and Tables

**Table 1:** Affinity sequences for Del.

Aptamers	Sequence of Aptamers (5′→3′)	GC (%)	ΔG (KJ/mol)
Del-1	P1-AAGGTGGAGTTATAGGCTTACTGCCACGTC-P2	50	ΔG = −12.333
Del-2	P1-GTGGCCGGTCAAGGCGTGATAGAAGAGTAA-P2	44	ΔG = −6.908
Del-3	P1-GTCGTGAGTGTTACGCCCGCGGGGGCTGGT-P2	70	ΔG = −17.50
Del-4	P1-TCGGTTAGACATCGAATGCTATCGTGGACC-P2	50	ΔG = −4.605
Del-5	P1-TAGACGCCTGCAGGTAAATGAAAACCAAGA-P2	44	ΔG = −2.595
Del-6	P1-ACGACAGATCGAGGCCTTCACCAGTACAAC-P2	54	ΔG = −6.698
Del-7	P1-TGGTTCGCGGGTTTGGCGCGTCTGGTGGCA-P2	67	ΔG = −17.626
Del-8	P1-CAGGACGGGAAGTGTCCCGTCTAGAAACAC-P2	60	ΔG = −33.368
Del-9	P1-CGGGACTTGGGCGTTGCTGGTCGTCGAAAA-P2	57	ΔG = −1.297
Del-10	P1-GTCCCCCGCAGTTTATAATGTAAAGGGAAA-P2	44	ΔG = −6.908
Del-11	P1-GTGTCCACAGAAAAATTCCGTATGGGCTCC-P2	60	ΔG = −3.475
Del-12	P1-GAACCTGCTCGTCGAGACGAAAGGCTTTCC-P2	57	ΔG = −19.761
Del-13	P1-CGGCCAGAAGACGGGTAGAGCACGTACCAT-P2	60	ΔG = −11.513
Del-14	P1-ACGATACGGTGAGAAGTTGTAACTAACGCA-P2	44	ΔG = −1.925
Del-15	P1-AATCCTGTATCGACCTGGGCTGCTGCGTGG-P2	70	ΔG = −2.386

**Table 2:** Actual samples tested and their recoveries.

Sample	Added (ng·mL^−1^)	Mean found (ng·mL^−1^)	Recovery (%)	RSD (%, n = 3)
	0	0	0	0
Apple	500	510	102	0.13
	1000	1180	118	6.12
	0	0	0	0
Pears	500	370	74	2.23
	1000	760	76	3.00

**Table 3:** Comparison of our Del detection method with other previously reported methods.

Method	LOD (ng·mL^−1^)	Equipment Required	Cost of Detection	Time Required
GC-FID [33]	0.01	General Purpose GC System	high	2–3 h
HPLC [34]	1	HPLC System	high	1–3 h
ELISA [35]	1.1 ± 0.5	Spectrometer	high	0.5–1 h
SERS [36]	0.0316 × 10^−3^	Raman Microscope System	high	1–2 h
Electrochemical [37]	77.3	Potentiostat	medium	15 min
Colorimetric [38]	173	No	low	10–20 min
This work	54.57	No	low	10–30 min

## Data Availability

The data that support the findings of this study are available from the corresponding author upon reasonable request.

## Supplementary Materials

The following supporting information can be downloaded at https://www.mdpi.com/article/10.3390/s25072060/s1. Figure S1: The first group has three rings (Secondary structures of candidate aptamers for Del:Del-14,Del-11). Figure S2: The second group has 4 rings (Secondary structures of candidate aptamers for Del:Del-13,Del-10,Del-6,Del-1,Del-3,Del-2,Del-8,Del-7,Del-12). Figure S3. The third group has 5 rings (Secondary structures of candidate aptamers for Del:Del-9,Del-5). Figure S4: The fifth group has six rings (Secondary structures of candidate aptamers for Del:Del-4,Del-15). Figure S5: Phylogenetic tree of 15 affinity sequences. Family I (30.0%): Del-2, Del-8, Del-6,Del-13,Del-1,Del-14. Family II (23.3%): Del-11, Del-4, Del-12. Family III (13.4%): Del-5, Del-10, Del-7, Del-9. Family IV (10.0%): Del-3, Del-15. Figure S6: Color changes in different sample systems. (Sample 1: AuNPs; Sample 2: PDDA + AuNPs; Sample 3: Del-1 + PDDA + AuNPs; Sample 4: Del-1 + Del (0.5 μg·mL^−1^) + PDDA + AuNPs; Sample 5: Del-1+ Del (1.2 μg·mL^−1^) + PDDA + AuNPs; Sample 6: Del-1 + Del (3 μg·mL^-1^) + PDDA + AuNPs. Figure S7: Effect of different concentrations of Del on the color system. The concentration of Del was 0.1μg·mL^−1^, 0.2 ug·mL^−1^, 0.4 ug·mL^−1^,0.5 ug·mL^−1^,0.7 ug·mL^−1^,0.8 ug·mL^−1^,1.0 ug·mL^−1^,1.2 ug·mL^−1^,2.0 ug·mL^−1^,3.0 ug·mL^−1^,4.0 ug·mL^−1^,5.0 ug·mL^−1^, respectively.
