# Supplementary material for: The Screening of Aptamers and the Development of a Colorimetric Detection Method for the Pesticide Deltamethrin
Source: Sensors (Basel). 2025 Mar 26;25(7):2060. doi: 10.3390/s25072060 (PMC11991097; doi:10.3390/s25072060)
Supplement: Supplementary file 1 [file sensors-25-02060-s001.zip › sensors-3524554-supplementary.pdf]

Supplementary data

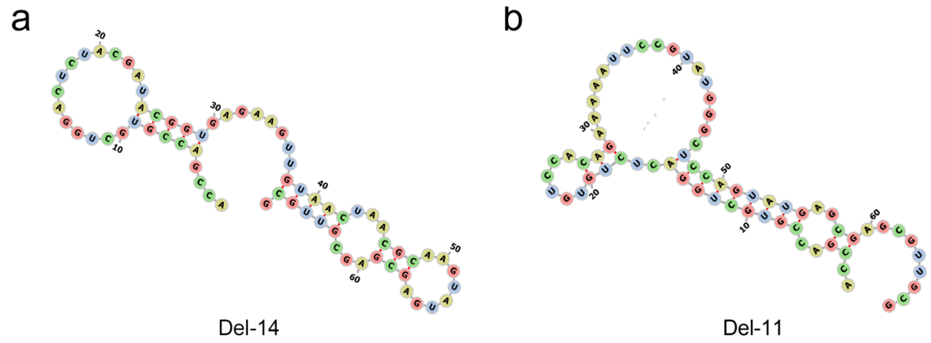

Figure S1. The first group has three rings (Secondary structures of candidate aptamers for Del:Del-14,Del-11)

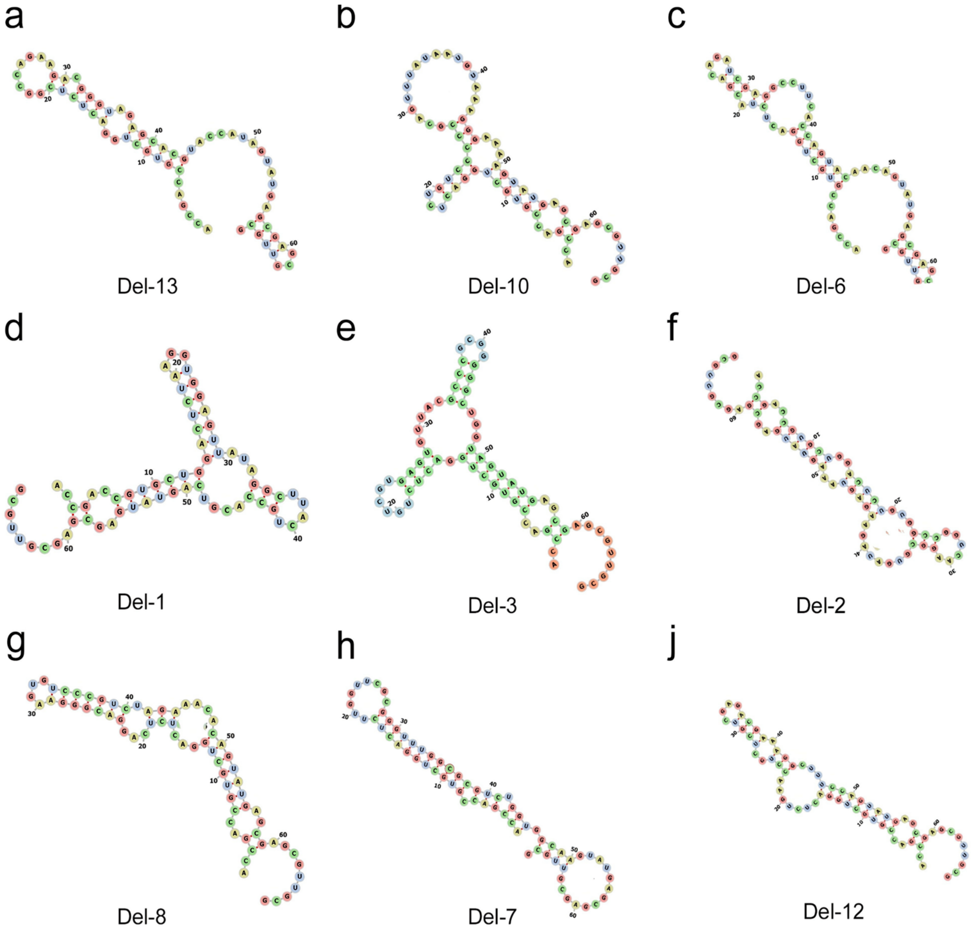

Figure S2. The second group has 4 rings (Secondary structures of candidate aptamers for Del:Del-13,Del-10,Del-6,Del-1,Del-3,Del-2,Del-8,Del-7,Del-12).

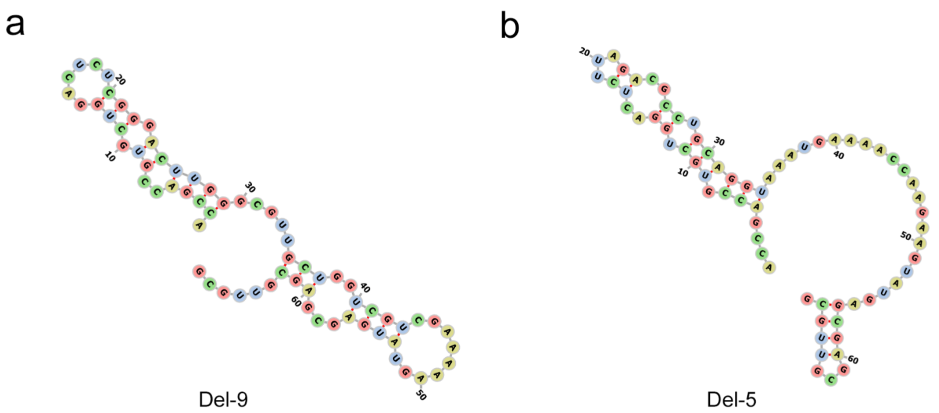

Figure S3. The third group has 5 rings (Secondary structures of candidate aptamers for Del：Del-9，Del-5)

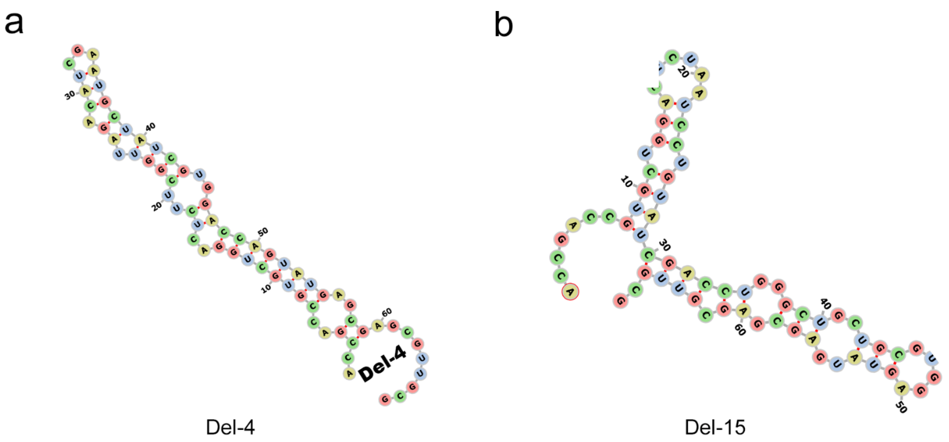

Figure S4. The fifth group has six rings (Secondary structures of candidate aptamers for Del：Del-4，Del-15)

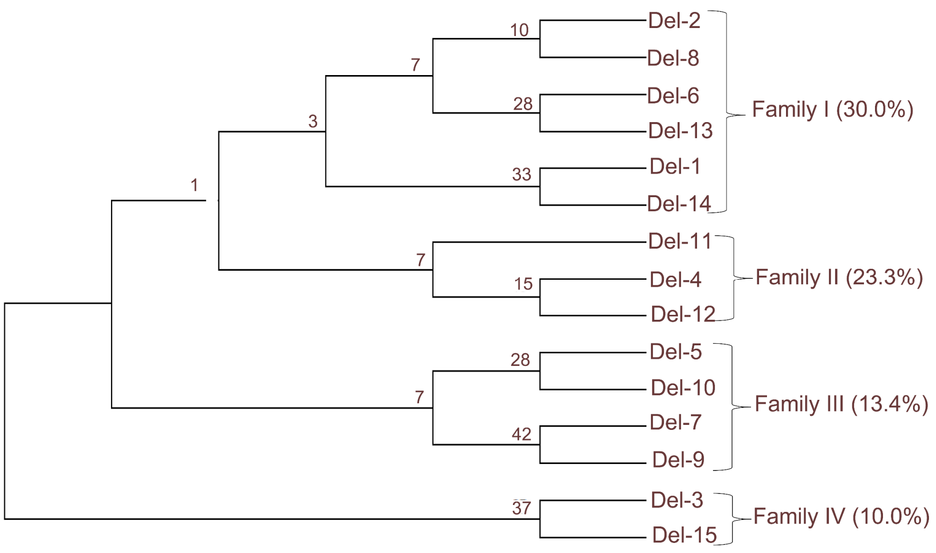

Figure S5. Phylogenetic tree of 15 affinity sequences. Family I (30.0%): Del-2, Del-8, Del-6,Del-13,Del-1,Del-14. Family II (23.3%): Del-11, Del-4, Del-12. Family III (13.4%): Del-5, Del-10, Del-7, Del-9. Family IV (10.0%): Del-3, Del-15.

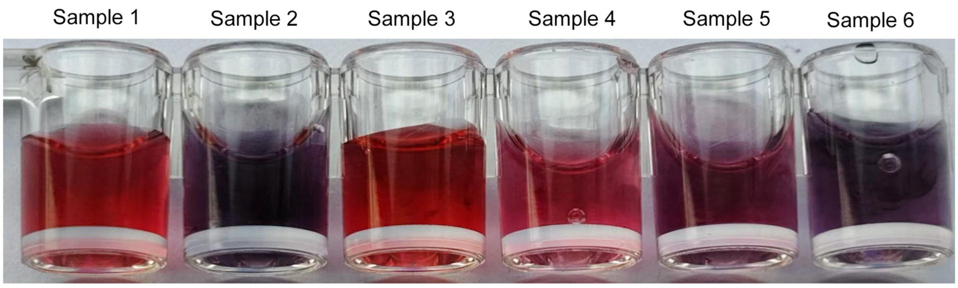

Figure S6. Color changes in different sample systems. (Sample 1: AuNPs; Sample 2: PDDA + AuNPs; Sample 3: Del-1 + PDDA + AuNPs; Sample 4: Del-1 + Del (0.5 μg·mL^{-1}) + PDDA + AuNPs; Sample 5: Del-1+ Del (1.2 μg·mL^{-1}) + PDDA + AuNPs; Sample 6: Del-1 + Del (3 μg·mL^{-1}) + PDDA + AuNPs.

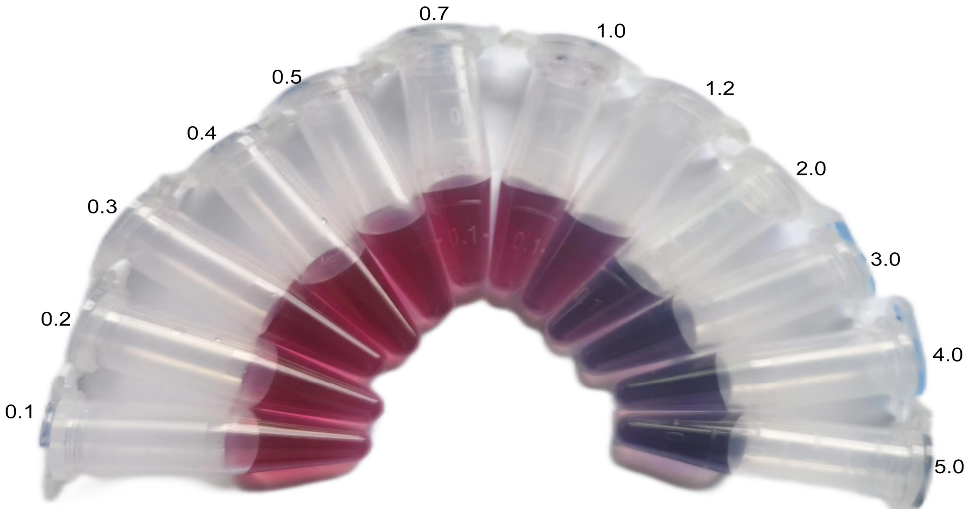

Figure S7. Effect of different concentrations of Del on the color system. The concentration of Del was 0.1μg·mL^{-1}, 0.2 ug·mL^{-1}, 0.4 ug·mL^{-1},0.5 ug·mL^{-1},0.7 ug·mL^{-1},0.8 ug·mL^{-1},1.0 ug·mL^{-1},1.2 ug·mL^{-1},2.0 ug·mL^{-1},3.0 ug·mL^{-1},4.0 ug·mL^{-1},5.0 ug·mL^{-1}, respectively.
